# Giants

**Published:** 1870-07

**Authors:** 


### GIANTS.

Pliny relates that, in the time of Cladius Cæsar there was a man, named Gabburus, brought by that emperor from Arabia to Rome, who was nine feet four inches high, "the tallest man that has been seen in our times."

In the reign of Augustus Cæsar, the tall forms of Rosis and Secundilla might have been seen, where bodies were preserved, in a museum in the Sallustian Gardens, and each of whom measured ten feet three inches in length.

The Emperor Maximus was nine feet high, and was in the habit of using his wife's bracelet as a thumb ring. His shoe was a foot longer than that of any other man, and he could draw a carriage which two oxen could not move. He ate usually forty pounds weight of flesh, and drank six gallons of wine every day.

Josephus tells of Eleazer, a Jew, a giant over ten feet high, who was one of the hostages whom the King of Persia sent to Rome after a peace.

Plot, in his "Oxfordshire," 1676, says, that a skeleton, seventeen feet high, was then to be seen in the town hall, in Lucerne. It had been found under an oak in Willison, near the village of Reyden.—*Record.*

